# ATP attenuates olaparib-induced gastric damage in rats

**DOI:** 10.3389/fphar.2026.1847232

**Published:** 2026-06-22

**Authors:** Mesud Fakirullahoglu, Ayetullah Temiz, Mustafa Yeni, Esra Tuba Sezgin, Gulce Naz Yazici, Durdu Altuner, Mine Gulaboglu, Halis Suleyman

**Affiliations:** 1 Department of General Surgery, Erzurum City Hospital, Erzurum, Türkiye; 2 Anesthesia Program, Vocational School of Health Services, Erzincan Binali Yıldırım University, Erzincan, Türkiye; 3 Department of Histology and Pathology, Faculty of Medicine, Erzincan Binali Yıldırım University, Erzincan, Türkiye; 4 Department of Pharmacology, Faculty of Medicine, Erzincan Binali Yıldırım University, Erzincan, Türkiye; 5 Department of Biochemistry, Faculty of Pharmacy, Ataturk University, Erzurum, Türkiye

**Keywords:** ATP, gastric injury, gastric mucosa, histopathology, olaparib, oxidative stress, PARP inhibitor

## Abstract

**Background:**

Olaparib, a poly (ADP-ribose) polymerase (PARP) inhibitor widely used in cancer therapy, has been associated with gastrointestinal adverse effects; however, the underlying mechanisms remain unclear. Previous studies have suggested that oxidative stress may contribute to olaparib-induced tissue injury.

**Objective:**

This study aimed to investigate the protective effects of adenosine triphosphate (ATP) against olaparib-induced gastric damage in rats.

**Methods:**

Animals were divided into healthy control (HG), olaparib alone (OLPG), ATP alone (ATPG), and ATP + olaparib (ATOLG) groups. Gastric injury was evaluated by macroscopic assessment of ulcer area and ulcer number. Histopathological alterations were examined using hematoxylin–eosin (H&E) staining, and oxidative stress status was assessed by measuring lipid peroxidation and antioxidant defense parameters.

**Results:**

Olaparib administration induced marked gastric mucosal injury, characterized by increased ulcer area and ulcer number, severe histopathological damage, and pronounced oxidative stress. In contrast, ATP co-administration significantly attenuated gastric injury, as evidenced by reduced ulcer area and ulcer number, improved histopathological architecture, and restoration of oxidative balance. Compared with the OLPG group, the ATOLG group showed significant improvement in macroscopic, histopathological, and oxidative stress parameters.

**Conclusion:**

ATP administration attenuated olaparib-induced gastric damage and improved oxidative stress parameters in rats. Although the underlying mechanisms were not directly investigated in the present study, the obtained findings provide preliminary experimental evidence suggesting that ATP may have protective potential against olaparib-induced gastric injury.

## 1 Introduction

Olaparib is an orally administered PARP inhibitor used in the treatment of recurrent ovarian cancer, metastatic breast cancer, and prostate cancer (Bochum et al., 2018) Olaparib has been approved as a first-line maintenance therapy in adults with advanced ovarian cancer who have shown a complete or partial response to first-line platinum-based chemotherapy (Paik, 2021). Adverse effects observed in patients using olaparib include diarrhea, vomiting, decreased appetite, dyspepsia, taste disturbances, dizziness, headache, back pain, urinary tract infections, shortness of breath, and cough. The most common adverse effects are hematological and gastrointestinal (GI) toxicities (Jin et al., 2025). Studies on PARP inhibitors have shown that olaparib is associated with a significantly increased risk of GI events (Sun et al., 2021). Moreover, the literature reports that olaparib causes oxidative damage by increasing malondialdehyde (MDA) levels in lipids and carbonyl levels in proteins in a tissue-dependent manner (Stefan et al., 2024). In a recent investigation, it was emphasized that olaparib triggers mitochondrial dysfunction in human ovarian cancer cells by increasing levels of reactive oxygen species (ROS) and decreasing antioxidant levels. Moreover, treatment with olaparib has been shown to result in reductions in complex I and complex IV activities and ATP production (Gao et al., 2025). Conversely, preclinical and clinical studies have not provided clear and consistent information explaining the pathogenesis of the adverse effects of olaparib on the gastrointestinal system. These findings from the literature suggest that oxidative stress, mitochondrial dysfunction, and ATP depletion may play a role in the development of adverse effects associated with olaparib treatment. However, the role of ATP depletion in olaparib-induced gastric injury has not been directly demonstrated and should be considered a hypothesis that requires further investigation. Therefore, ATP was evaluated in the present study as a potential protective agent against olaparib-induced gastric injury. ATP, whose protective effect against olaparib-induced gastric damage we aim to investigate, is the primary energy source for cellular use (Gao et al., 2025).

At the molecular level, ATP is a nucleoside triphosphate composed of a nitrogenous base, a ribose sugar, and three phosphate groups ATP hydrolysis is essential for a wide range of cellular functions, including signal transduction and DNA/RNA synthesis (Dunn and Grider, 2020). As is well known, mitochondria are the main source of cellular ROS. This ROS attack leads to mitochondrial damage, impairment of oxidative phosphorylation, and consequently a decrease in ATP synthesis (Bulut and Süleyman, 2024). Previous studies have shown that oxidative stress is associated with ATP depletion (Nankivell et al., 2025). Furthermore, ATP is known to participate in the synthesis of ROS-scavenging and detoxifying antioxidants and to provide energy for the synthesis of low-molecular-weight antioxidants (Saquet et al., 2000; Yi et al., 2010).

These data from the literature indicate that ATP may be beneficial in the treatment of gastric damage that may occur due to olaparib use Also, no studies were found in the literature investigating the effect of ATP in the treatment of olaparib-induced gastric damage. Thus, the aim of our study is to investigate the effect of ATP against olaparib-induced gastric injury in rats.

## 2 Materials and methods

### 2.1 Animals

Male albino Wistar rats weighing between 270 and 284 g were used in the experiment. A total of 24 male **r**a**t**s were obtained from the Experimental Animals Application and Research Center of Erzincan Binali Yildirim University. Prior to the experiment, all animals were housed in groups (n = 6) under controlled conditions at a temperature of 21 °C–22 °C, relative humidity of 30%–70%, and a 12 h light/12 h dark cycle, with free access to water and standard pellet feed. All animal procedures were approved by the Local Ethics Committee for Animal Experiments of Erzincan Binali Yildirim University (Erzincan, Turkey) (Approval No. 2026/01; Date: 29 January 2026) and were conducted in accordance with the European Parliament Directive 2010/63/EU for the protection of animals used for scientific purposes. The study was also performed in compliance with the ARRIVE guidelines (Du Sert et al., 2020).

### 2.2 Chemicals

Olaparib (150 mg tablets; AstraZeneca, Istanbul, Turkey), ketamine hydrochloride (Pfizer, USA), xylazine hydrochloride (Bayer, Germany) and ATP (Zdorove Narodu, Ukraine) were used in the experiment.

### 2.3 Experimental design and grouping

#### 2.3.1 Experimental design

The sample size was determined to use the minimum number of animals required in accordance with the principles of the 3Rs (Replacement, Reduction, and Refinement) (Kiani et al., 2022). The sample size (n = 6 per group) was also based on previously published experimental studies in which similar group sizes were sufficient to achieve statistical significance. Throughout the experimental period, animals were monitored for predefined humane endpoint criteria, including slumped posture, reduced mobility, and injuries caused by other animals. These criteria were prospectively defined to allow removal of animals if necessary; however, no animals or data points were excluded during the experiment or data analysis. Randomization was performed using a random number table to generate the allocation sequence. To minimize potential confounding factors, cages and animals were coded and assigned identification numbers, and all experimental procedures and assessments were conducted using these codes.

#### 2.3.2 Experimental groups

The animals were randomly assigned to four groups: healthy control (HG), olaparib alone (OLPG), ATP alone (ATPG), and ATP + olaparib (ATOLG).

### 2.4 Experiment procedure

ATP was administered intraperitoneally (i.p.) at a dose of 5 mg/kg to the ATPG (n = 6) and ATOLG (n = 6) groups, based on previously reported experimental studies. Distilled water was administered as a vehicle to the HG (n = 6) and OLPG (n = 6) groups. Olaparib tablets (150 mg/tablet) were freshly suspended in distilled water prior to administration. To ensure accurate dosing, the concentration of the suspension was adjusted according to the body weight of each animal. One hour after the administration of ATP or vehicle, olaparib was administered orally by gastric gavage at a dose of 100 mg/kg twice daily to the ATOLG and OLPG groups. The olaparib dose and twice-daily administration schedule were selected based on previously published pharmacokinetic studies reporting the use of olaparib at 100 mg/kg BID in experimental models (Sun et al., 2018). ATP was administered once daily via the i. p. route. This procedure was continued for 2 weeks. At the end of the experimental period, animals were anesthetized using an i. p. injection of ketamine (80 mg/kg) and xylazine (10 mg/kg) and gastric tissues were excised. Adequate depth of anesthesia was confirmed by the absence of reflex responses prior to any experimental procedure. The stomach tissues were first evaluated macroscopically and then subjected to biochemical and histopathological examinations. Following macroscopic evaluation, the gastric tissues were divided into separate portions for biochemical and histopathological analyses. Tissue samples designated for histopathological examination were fixed in 10% buffered formalin, whereas the remaining gastric tissues were used for biochemical analyses. Macroscopic, biochemical, and histopathological findings obtained from the experiment were compared and evaluated among the groups. For macroscopic evaluation, the stomach tissues were opened and examined for visible gastric lesions. The ulcerated regions were measured on millimeter paper, and the total ulcer area was expressed in mm^2^. Ulcer number was defined as the total number of macroscopically visible ulcerative lesions on the gastric mucosa. To reduce observer bias, macroscopic evaluations were performed using coded samples.

### 2.5 Biochemical analysis

#### 2.5.1 Preparation of samples

After measuring the weights of the samples, the tissues were cut into small pieces, flash-frozen in liquid nitrogen, and homogenized using a mortar and pestle. The homogenates were vortexed for 10 s with phosphate-buffered saline (PBS; pH 7.4) at a ratio of 1:10 (w/v), centrifuged at 10,000 rpm for 20 min, and the supernatants were collected. The samples were stored at −80 °C until analysis.

#### 2.5.2 Determination of MDA, tGSH, SOD, CAT and protein in gastric tissue

Tissue MDA, tGSH, and SOD levels were measured using rat-specific enzyme-linked immunosorbent assay (ELISA) kits according to the manufacturers’ instructions (product numbers 10009055, 703,002, and 706,002, respectively; Cayman Chemical Company, Ann Arbor, MI, USA). CAT activity was determined according to the method described by Goth (Goth, 1991). Tissue protein levels were determined using the method described by Bradford (Bradford, 1976).

### 2.6 Histopathological analysis

Tissue samples were fixed in 10% buffered formalin for 72 h, washed under running tap water for 24 h, and dehydrated through a graded ethanol series (70%–100%). After clearing in xylene, tissues were embedded in paraffin. Sections of 4–5 µm thickness were cut and stained with hematoxylin and eosin (H&E) (Bancroft and Gamble, 2008). Histological sections were examined and photographed using a light microscope (Olympus Inc., Tokyo, Japan) equipped with DP2-SAL imaging software. From serial sections, one central and five peripheral fields were selected for semi-quantitative evaluation. Gastric tissue damage was assessed based on epithelial damage, vascular congestion, inflammatory cell infiltration, and mucosal edema. Each parameter was graded on a semi-quantitative scale from 0 to 3 (0 = none, 1 = mild, 2 = moderate, 3 = severe). All evaluations were performed by a pathologist blinded to the experimental groups.

### 2.7 Statistical analysis

Biochemical data obtained from the experiments were expressed as mean ± SD. Biochemical parameters and macroscopic ulcer parameters (ulcer area and ulcer number) were analyzed using one-way analysis of variance (ANOVA) followed by Tukey’s post-hoc test for multiple comparisons. Semi-quantitative histopathological scores (hyperemia, edema, epithelial damage, vascular congestion, and inflammatory cell infiltration) were analyzed using the nonparametric Kruskal–Wallis test, followed by the Mann–Whitney U test for pairwise comparisons. All statistical analyses were performed using SPSS software (IBM SPSS Statistics for Windows, Version 20.0; IBM Corp., Armonk, NY, USA). A value of p < 0.05 was considered statistically significant.

## 3 Results

### 3.1 Macroscopic findings of gastric tissue

As shown in Figure 1, no macroscopic gastric damage was detected in the HG, ATPG, or ATOLG groups. In contrast, the OLPG group showed marked hyperemia and multiple ulcerative lesions. Accordingly, Table 1 demonstrates that the olaparib-alone group exhibited a significantly increased mean gastric ulcer area (4.3 mm2) and mean ulcer number (3.16) compared with the HG and ATPG groups (*p* < 0.05).

**FIGURE 1:**
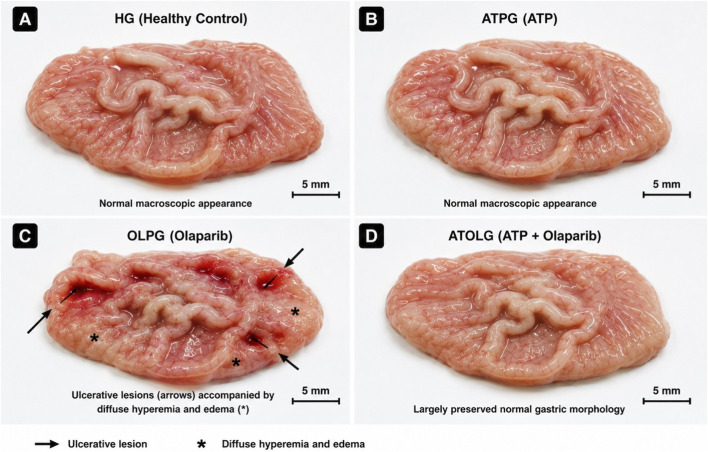
Macroscopic evaluation of gastric tissues from the HG, ATPG, OLPG, and ATOLG groups. **(A,B)** The gastric tissues of the HG and ATPG groups exhibited a normal macroscopic appearance. **(C)** In the OLPG group, ulcerative lesions (arrows) accompanied by diffuse hyperemia and edema (*) were observed. **(D)** The ATOLG group showed a largely preserved normal gastric morphology. Abbreviations: HG: healthy control group; ATPG: adenosine triphosphate group; OLPG: olaparib group; ATOLG: ATP + olaparib group.

**TABLE 1:** Macroscopic evaluation of gastric mucosal injury.

Groups	Ulcer area (mm^2^)	Ulcer number
HG	0	0
ATPG	0	0
OLPG	4.3 ± 1.37	3.17 ± 1.47
ATOLG	0	0

Data are expressed as mean ± standard deviation (SD) for ulcer area and ulcer number. Statistical analysis was performed using one-way ANOVA, followed by Tukey’s *post hoc* test (n = 6 per group). Abbreviations: HG, healthy control group; ATPG, adenosine triphosphate group; OLPG, olaparib group; ATOLG, ATP + olaparib group.

### 3.2 Biochemical findings

#### 3.2.1 MDA and tGSH levels in gastric tissue

MDA levels were evaluated using one-way ANOVA. The analysis revealed a statistically significant difference among the groups (F = 189.75, p < 0.001). Tukey’s post-hoc test showed that MDA levels in the OLPG were significantly higher than those in the HG, ATPG, and ATOLG (p < 0.001). In the ATOLG, MDA levels were significantly lower compared with the OLPG (p < 0.001). No statistically significant differences in MDA levels were observed between the HG and the ATPG or ATOLG (p > 0.05) (Figure 2A; Table 2).

**FIGURE 2:**
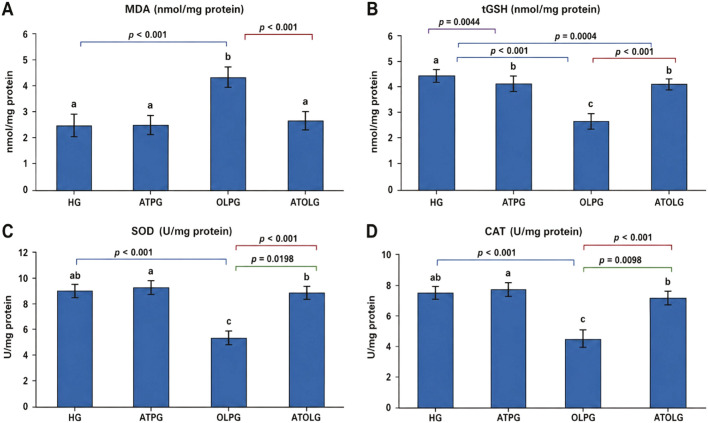
Effects of treatments on gastric oxidative stress parameters: **(A)** MDA, **(B)** tGSH, **(C)** SOD, and **(D)** CAT. Data are expressed as mean ± SD (n = 6). Statistical analysis was performed using one-way ANOVA followed by Tukey’s *post hoc* test. Different lowercase letters indicate statistically significant differences between groups (p < 0.05). Groups sharing at least one common letter are not significantly different, whereas groups with different letters differ significantly. Exact p-values for selected pairwise comparisons are shown above the comparison brackets. Abbreviations: HG: healthy control group; ATPG: adenosine triphosphate group; OLPG: olaparib group; ATOLG: ATP + olaparib group; MDA: malondialdehyde; tGSH: total glutathione; SOD: superoxide dismutase; CAT: catalase.

**TABLE 2:** Effects of ATP on oxidative stress in olaparib induced ulcerative gastric injury in rats.

Groups	MDA (nmol/mg protein)	tGSH (nmol/mg protein)	SOD (U/mg protein)	CAT (U/mg protein)
HG	2.46 ± 0.24	4.53 ± 0.11	9.11 ± 0.24	7.63 ± 0.23
ATPG	2.53 ± 0.14	4.26 ± 0.20	9.38 ± 0.20	7.78 ± 0.30
OLPG	4.38 ± 0.11	2.67 ± 0.13	5.36 ± 0.15	4.43 ± 0.17
ATOLG	2.71 ± 0.12	4.17 ± 0.06	9.00 ± 0.22	7.27 ± 0.27
Group comparisons	p values	​	​	​
HG vs. ATPG	> 0.05	0.0044	> 0.05	0.7296
HG vs. OLPG	< 0.001	< 0.001	< 0.001	< 0.001
HG vs. ATOLG	> 0.05	0.0004	> 0.05	0.0872
ATPG vs. OLPG	< 0.001	< 0.001	< 0.001	< 0.001
ATPG vs. ATOLG	> 0.05	0.6231	0.0198	0.0098
OLPG vs. ATOLG	< 0.001	< 0.001	< 0.001	< 0.001
F value	189.75	238.74	524.67	243.99
df (df1/ df2)	3/20	3/20	3/20	3/20
P	< 0.001	< 0.001	< 0.001	< 0.001

Results are expressed as mean ± SD., Statistical analysis was performed using one-way ANOVA, followed by Tukey’s post-hoc test for multiple comparisons (n = 6). Statistical significance was set at *p* < 0.05. Abbreviations: HG: healthy control group; ATPG: adenosine triphosphate group; OLPG: olaparib group; ATOLG: ATP + olaparib group; MDA: malondialdehyde; tGSH: total glutathione; SOD: superoxide dismutase; CAT: catalase.

Total glutathione (tGSH) levels were analyzed using one-way ANOVA, and a statistically significant difference was detected among the groups (F = 238.74, *p* < 0.001). Tukey’s post-hoc analysis demonstrated that tGSH levels in the olaparib group were significantly lower than those in the HG, ATPG, and ATOLG (*p* < 0.001). In addition, the ATPG showed a significant difference compared with the HG (*p* = 0.0044), and the ATOLG was also significantly different from the HG (*p* = 0.0004). No significant difference was observed between the ATPG and ATOLG (*p* = 0.6231) (Figure 2B; Table 2).

#### 3.2.2 SOD and CAT levels in gastric tissue

Superoxide dismutase (SOD) levels were analyzed using one-way ANOVA, which revealed a statistically significant difference among the groups (F = 524.67, *p* < 0.001). Tukey’s post-hoc analysis indicated that SOD levels in the OLPG were significantly lower than those in the HG, ATPG, and ATOLG (*p* < 0.001). A significant difference was also observed between the ATPG and ATOLG (*p* = 0.0198). However, no significant differences were detected between the HG and the ATPG or ATOLG (*p* > 0.05) (Figure 2C; Table 2).

Catalase (CAT) activity was analyzed using one-way ANOVA, which revealed a statistically significant difference among the groups (F = 243.99, p < 0.001). Tukey’s post-hoc test demonstrated that CAT activity in the OLPG was significantly lower than that in the HG, ATPG, and ATOLG (p < 0.001 for all comparisons). A significant difference was also observed between the ATPG and ATOLG (p = 0.0098). However, no statistically significant differences were detected between the HG and ATPG (p = 0.7296) or between the HG and ATOLG (p = 0.0872) (Figure 2D; Table 2).

### 3.3 Histopathological findings

Histopathological examination of gastric tissue sections from the HG revealed that the tunica mucosa, surface epithelium, gastric glands, and overall gastric wall layering preserved a normal histological architecture (Figure 3A). Similarly, in the ATPG, the surface epithelium, glandular structure, and blood vessels appeared histologically normal, with no evidence of tissue injury (Figure 3B). In contrast, gastric tissue sections from the OLPG exhibited marked histopathological alterations. These included focal detachment of the surface epithelium with luminal debris formation, a reduction in glandular invaginations, widening of the glandular neck regions, and mild to moderate edema in the glandular base. Additionally, moderate dilation and congestion of capillary vessels were observed. Notably, marked inflammatory cell infiltration was detected in the perivascular areas and connective tissue adjacent to the glandular bases (Figure 3C). Evaluation of gastric tissues from the ATOLG demonstrated substantial histological improvement compared with the OLPG. The overall mucosal architecture remained largely preserved, although mild vascular congestion, edema, and inflammatory cell infiltration were observed. (Figure 3D). Semi-quantitative histopathological scoring demonstrated a significant increase in gastric tissue damage in the OLPG compared with the HG and ATPG groups (p < 0.05). While no significant histopathological alterations were observed in the HG and ATPG groups, the OLPG exhibited markedly elevated scores for epithelial damage, edema, vascular congestion, and inflammatory cell infiltration. In contrast, the ATOLG group showed mild histopathological alterations, with significantly lower damage scores compared with the OLPG (p < 0.05). These findings indicate that combined ATP and olaparib treatment markedly attenuated olaparib-induced gastric injury (Table 3).

**FIGURE 3:**
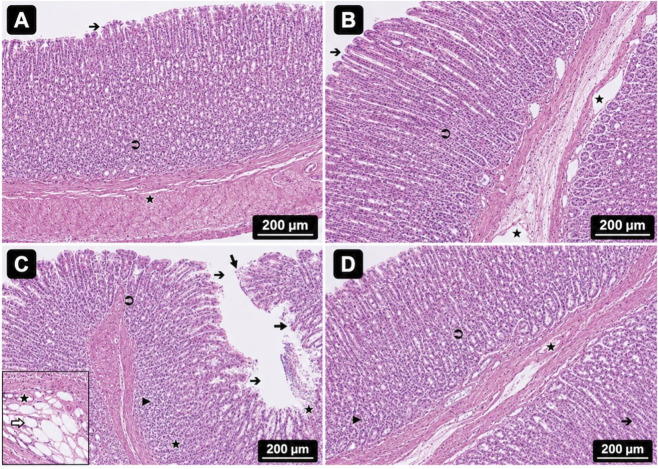
Representative histopathological images (H&E, 200×) of gastric tissues from **(A)** HG, **(B)** ATPG, **(C)** OLPG, and **(D)** ATOLG groups. The images shown are representative sections selected from each experimental group and reflect the group-level histopathological findings and semi-quantitative scoring results. **(A)** HG group shows normal gastric mucosa with surface epithelium (→), normal gastric glands (➲), and blood vessel (★), without evident pathological changes. **(B)** ATPG group shows a histological appearance similar to that of the HG group, with preserved surface epithelium (→), normal gastric glands (➲), and blood vessel (★) without remarkable histopathological alterations. **(C)** OLPG group shows ulcerative lesions and erosion (→) with diffuse hyperemia and edema in the submucosa (★), accompanied by vascular congestion (➲) and inflammatory cell infiltration (►). Inset: submucosal edema (⇨). **(D)** ATOLG group shows largely preserved mucosal architecture; with intact surface epithelium (→), mild residual inflammatory cell infiltration (►), mild vascular congestion (➲) and mild submucosal edema (★) without ulceration. Abbreviations: HG: healthy control group; ATPG: adenosine triphosphate group; OLPG: olaparib group; ATOLG: ATP + olaparib group.

**TABLE 3:** Histopathological injury scores of gastric tissue in experimental groups.

Groups	Epithelial damage	Glandular degeneration	Edema	Vascular congestion	Inflammatory cell infiltration
HG	0.00 (0–0)	0.00 (0–0)	0.00 (0–0)	0.00 (0–0)	0.00 (0–0)
ATPG	0.00 (0–0)	0.00 (0–0)	0.00 (0–0)	0.00 (0–0)	0.00 (0–0)
OLPG	2.00 (2–3)^*^	2.00 (1–2)^*^	2.00 (1–2)^*^	2.00 (2–3)^*^	3.00 (2–3)^*^
ATOLG	1.00 (0–1)^#^	1.00 (0–1)^#^	1.00 (0–1)^#^	1.00 (1–2)^#^	1.00 (0–1)^#^

Histopathological scoring was performed on a scale of 0–3 (0 = no damage, 1 = mild, 2 = moderate, 3 = severe). Data are expressed as median (min–max). Statistical analysis was performed using the Kruskal–Wallis test followed by the Mann–Whitney U test (n = 6).

^*^ p < 0.05 vs. HG.

^#^ p < 0.05 vs. OLPG. Abbreviations: HG: healthy control group; ATPG: adenosine triphosphate group; OLPG: olaparib group; ATOLG: ATP + olaparib group.

## 4 Discussion

In the present study, the effects of ATP against olaparib-induced ulcerative gastric injury in rats were evaluated using macroscopic, biochemical, and histopathological methods. The present results indicate that olaparib administration causes marked ulcerative damage in rat gastric tissue, which is associated with oxidative stress, impaired antioxidant defense, and inflammatory responses. When the macroscopic, biochemical, and histopathological findings were evaluated together, it was evident that olaparib activates a multifactorial damage mechanism in the gastric mucosa (Suleyman et al., 2010).

PARP inhibitors exert their anticancer effects primarily by suppressing DNA repair mechanisms in tumor cells However, PARP enzymes are known to play important roles not only in malignant cells but also in normal tissues, including DNA repair, cellular stress responses, and regulation of mitochondrial function. Consequently, the occurrence of toxicity in normal tissues following PARP inhibitor treatment is biologically plausible (Basu et al., 2012). In the present study, the marked hyperemia and ulcerative lesions observed in the gastric mucosa of the olaparib-treated group support this concept. The gastrointestinal system is highly susceptible to oxidative stress due to its high rate of cellular turnover and intense mitochondrial activity. The significant increase in MDA levels, a marker of lipid peroxidation (LPO), observed in gastric tissue following olaparib administration indicates that cell membranes are exposed to ROS-mediated damage. Consistent with our findings, previous studies have reported that PARP inhibitors induce oxidative damage in normal tissues by increasing MDA and protein carbonyl levels (Stefan et al., 2024). Increased MDA levels resulting from LPO disrupt cellular integrity and weaken mucosal barrier function, thereby facilitating the development of gastric ulcers (Suleyman et al., 2010). In the present study, the increase in ulcer area and number in the olaparib group is consistent with the biochemical findings and aligns with studies demonstrating a direct relationship between oxidative stress and structural damage in the gastric mucosa (Polat et al., 2011). It is well established that endogenous antioxidant defense systems are activated in response to increased oxidative stress. Despite this, excessive oxidant production can overwhelm these defense mechanisms, leading to antioxidant depletion (Kisaoglu et al., 2013). In our study, the significant reduction in tGSH levels in the olaparib-treated group indicates depletion of the endogenous GSH pool, which plays a critical role in ROS detoxification. This decrease in GSH levels renders cells more vulnerable to oxidative damage (Dickins et al., 2002). Likewise, suppression of the antioxidant enzymes SOD and CAT following olaparib administration suggests uncontrolled ROS accumulation. While SOD converts superoxide anions into hydrogen peroxide, CAT catalyzes the decomposition of hydrogen peroxide into water and oxygen. Reduced activity of these enzymes exacerbates oxidative stress and deepens tissue injury (Valko et al., 2007). Previous studies have also shown that PARP inhibitors impair mitochondrial function, increase ROS production, and disrupt mitochondrial dynamics. In particular, olaparib has been demonstrated to reduce mitochondrial complex I and IV activities and decrease ATP production (Gao et al., 2025).

A notable finding of this study is that ATP administration markedly attenuated olaparib-induced gastric mucosal damage. ATP treatment prevented ulcer formation, suppressed the increase in MDA levels, and reversed the decline in antioxidant parameters in gastric tissue. These findings are consistent with previous reports demonstrating that ATP exerts strong cytoprotective effects on mucosal tissues. In addition, our results are supported by studies showing that ATP promotes healing of stress-induced gastric ulcers and exhibits gastroprotective effects (Salcan et al., 2025). ATP is known to be essential for antioxidant defense, and its deficiency renders tissues more susceptible to injury (Bulut and Süleyman, 2024). Previous studies have also demonstrated a close association between oxidative stress and ATP depletion (Nankivell et al., 2025). Extracellular ATP is known to mediate its biological effects through purinergic receptors (P2X/P2Y) (Patrakeeva, 2025)**.** However, the involvement of these pathways was not evaluated in the present study.

The biochemical findings obtained in this study were in close agreement with the histopathological observations. Histopathological examination of the olaparib-treated group revealed mucosal degeneration, vascular dilation, congestion, and edema. In contrast, preservation of mucosal architecture, attenuation of inflammatory cell infiltration, and reduction of edema in the ATP + olaparib group were consistent with the biochemical results. Although no macroscopic gastric lesions were observed in the ATOLG group, histopathological evaluation revealed mild alterations, including edema, vascular congestion, and inflammatory cell infiltration. These findings indicate that ATP effectively prevented gross ulcer formation and substantially alleviated tissue injury; however, complete restoration of the microscopic architecture of the gastric tissue was not achieved. Therefore, the protective effect of ATP should be interpreted not as a complete prevention of olaparib-induced gastric damage, but rather as a significant attenuation of the injury. These findings support previous studies demonstrating that ATP exerts protective effects not only at the biochemical level but also on tissue integrity (Salcan et al., 2025). The presence of intense polymorphonuclear cell infiltration accompanying structural disruption in the olaparib group indicates that oxidative stress triggers inflammatory responses. It is well established that ROS increase endothelial permeability, facilitate inflammatory cell migration, and promote tissue edema. This mechanism is consistent with the histological alterations observed in the present study (Mittal et al., 2014).

This study has several limitations. Firstly, the research was conducted using healthy male rats rather than tumor-bearing animals, and therefore the findings cannot be directly extrapolated to cancer patients receiving long-term olaparib treatment. Furthermore, the inclusion of only male rats in the experimental design may limit the generalizability of the findings. Future studies involving larger sample sizes and both sexes may allow a more comprehensive evaluation of the protective effects of ATP. In addition, the research was conducted using an experimental rat model, and the findings cannot be directly extrapolated to humans. Differences in the physiological and biochemical characteristics of rat and human gastric mucosa necessitate further clinical studies for translation into clinical practice. Only a single dose and treatment duration of olaparib were evaluated. The lack of different dosing regimens, treatment durations, and chronic exposure models limits the assessment of dose–response relationships and long-term effects on gastric tissue. Similarly, the use of a single ATP dose precluded determination of the optimal protective dose range. In addition, exogenous ATP is inherently unstable and can be rapidly degraded by enzymes such as CD39 and CD73, limiting its bioavailability. ATP exerts its biological effects mainly through purinergic signaling pathways. Moreover, tissue ATP levels were not directly measured, which limits confirmation of ATP depletion and weakens mechanistic interpretation. It also remains unclear whether the observed antioxidant effects are mediated directly by ATP or indirectly through endogenous pathways. Since functional parameters such as mucus production, gastric acid secretion, and barrier integrity were not evaluated, the assessment of functional recovery is limited. In addition, the absence of comparison with established gastroprotective agents restricts the interpretation of the comparative efficacy of ATP. Finally, evaluation of the inflammatory response was largely limited to histopathological findings, and the lack of molecular analysis of proinflammatory cytokines reduced the depth of mechanistic insight.

## 5 Conclusion

In conclusion, the present study demonstrates that olaparib administration is associated with ulcerative gastric injury accompanied by increased oxidative stress, impaired antioxidant defense, and histopathological alterations in gastric tissue. ATP administration significantly attenuated these changes and preserved gastric mucosal integrity. Although the underlying mechanisms were not directly investigated, the findings provide preliminary experimental evidence that ATP may exert protective effects against olaparib-induced gastric injury. Further experimental studies, including mechanistic investigations and functional gastric assessments, as well as well-designed clinical studies, are required to confirm these findings and determine their potential translational relevance.

## Data Availability

The original contributions presented in the study are included in the article/supplementary material, further inquiries can be directed to the corresponding author.
